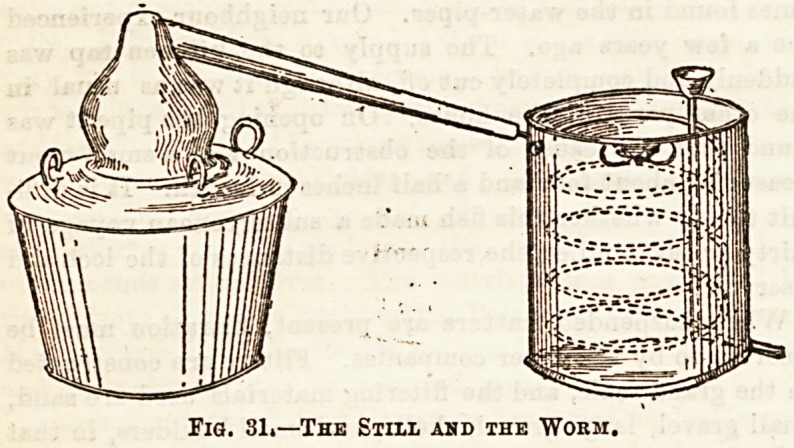# "The Hospital" Nursing Mirror

**Published:** 1896-07-25

**Authors:** 


### "The Hospital" Nursing Mirror.

Being the Extra Nursing Supplement of "The Hospital" Newspaper.

[Contributions for this Supplement should be addressed to the Editor, The Hospital, 428, Strand, London, W.C., and should have the word "Nursing" plainly written in left-hand top corner of the envelope.]

### News from the Nursing World.

### A GIFT FROM THE QUEEN.

Princess Louise has sent a present of some beautiful pictures to the Royal National Hospital for Consumption at Ventnor in the name of the Queen. It is to be hoped that this action of the Royal Family will stimulate an increased interest in this most useful institution now that it is to be so closely associated with the memory of Prince Henry of Battenberg by the erection of a block named after him. Already a large number of patients are awaiting admission to it.

### THE BAZAAR AT LANCASTER.

Lancaster now possesses a fine infirmary, but as is mostly the case when a charity is improved and expanded, the funds which sufficed before fall sadly short of what is necessary under the new conditions. This has been found to be the case at Lancaster, and so the friends of the infirmary in and around the town have been making special efforts to supply the special needs. Last week the Countess of Bective opened a grand bazaar, which lasted for three days. Great interest was taken in the event, and the employés of Lord Ashton and Sir Thomas Storey's mills alone contributed £100 in money and goods towards it. There were many attractive entertainments, and as the sale proceeded very briskly, we hope to hear of a substantial result. £4,000 is actually wanted.

### THE EXPENSES OF ADVERTISING.

On the one hand we hear that there is not enough work for nurses to do, and, on the other, that nurses are not to be had when required. Both statements are true, and both arise from mistaken notions. For union infirmaries and for district nursing the supply is very small. The unions, as a rule, ask too much of their nurses in the way of work, and for district nursing comparatively few nurses are trained as yet, and the pay is for the most part very small. Consequently, when a board of guardians advertise for a nurse, it is very general to suppose that they require someone who is willing to undertake the charge of as many as 60 beds with none but pauper assistance. It is not surprising to hear that advertising for two nurses for their infirmary cost a certain board of guardians not less than £20. The arrangements which render such expenditure necessary surely suggest the necessity of modification. The other source from which advertising columns are largely fed is the private nurse over 35 years of age. We should call this again the result of mistaken economy. For chronic cases, especially, the older nurse is by far the most suited. She has more experience, often the patience and even the strength which her younger sister has not attained, and an interview will usually reveal whether she is an active person or not. Yet because the conventional age of 35 has been passed her difficulties of securing a post become greater each time she is out of employment, until at last the larger portion of her earnings are spent in advertising. We hope to see the day when this unnecessary expenditure is much reduced by reasonable inducements to nurses being offered by all boards of guardians and by the education in public opinion to the fact that a capable, able-bodied woman is equal to nursing for at least 15 or 20 years longer than the present popular limit allows.

### JUNIUS S. MORGAN BENEVOLENT FUND.

The hon. secretary of this benevolent fund asks us to mention that the suggestion of the holder of Policy No. 1,976, that all nurses belonging to the Royal National Pension Fund for Nurses should subscribe annually the small sum of 1s. to their benevolent fund, is still being responded to. Policyholder No. 1,976 herself has taken a most active part in the suggested scheme, and a good number of contributions have been received through her. She has asked that these should be acknowledged through our columns, with which request we have pleasure in complying. Contributions of 2s. or 2s. 6d. have been received from A. L. M., J. K., T. S., A. C., B. M., M. J., E. F. H., O. W., J. D., E. M., C. J., E. O. H. The honorary secretary informs us that the balance of the Benevolent Fund is now so small that all but the most urgent cases have to be refused for the present.

### TOYNBEE NURSING GUILD.

This guild has just concluded a very busy session. Established in January of this year, it draws its membership mainly from students of ambulance classes for women at Toynbee Hall, and aims at rendering them capable of undertaking voluntary district nursing under the direction of existing nursing agencies. Copies of The Hospital and other magazines have been circulated each week, and the nucleus of a nursing library has been formed. Special monthly lectures have been given by Dr. Stephen Mackenzie on "Home Nursing," Dr. Eddowes on "Amateur Nurses," Dr. Dundas Grant on "Diseases of the Nose, Throat, and Ear," and by Mr. Rowland on the "New Photography." By the courtesy of Miss Lückes, matron of the London Hospital, a special course of lessons in bandaging has been given by Sister "Receiving Room" at Toynbee Hall. At the conclusion of the course an examination was held by Sister Morgan, of the hospital staff, and the candidates obtained marks varying from 55 to 92 per cent. of the total possible. The members subsequently asked Sister "Receiving Room" to accept a framed copy of Raphael's Sistine Madonna as a slight acknowledgment of her services. Further particulars can be obtained from the hon. secretaries, Miss M. M. Wills, or Mr. W. H. Winny, at Toynbee Hall, E.

### A PLEASANT EXCURSION.

On the afternoons of Thursday and Friday last the nurses of the Wolverhampton and South Staffordshire General Hospital were invited to spend a few hours at Castle Croft, the country residence of Mr. and Mrs. Twentyman. The nurses were driven to and from in a large brake, through the green country lanes, leaving the smoke and tall chimneys of the Black Country in the rear. On arriving they were received by Mrs. and the Misses Twentyman, who escorted them to the dining-room, where tea was awaiting them—the table laden with all kinds of delicacies and seasonable fruits. Tea being over, the nurses proceeded to roam through the grounds and gardens, accompanied by different members of the family, whilst those who were more energetic indulged in tennis or croquet. The nurses thoroughly enjoyed themselves, and were highly delighted with the kindness and hospitality which they received.

### CHICHESTER GUARDIANS AND PRIVATE NURSING.

The nursing arrangements at the Chichester Workhouse Infirmary have been recently much altered and improved. The Guardians at Chichester are evidently very progressive, and realising that both profit and prestige attaches to hospitals who send out private nurses, they suggested the scheme in connection with their infirmary to the Local Government Board. The Local Government Board probably preferring the tortoise method of progress, have not sanctioned this pioneer proposal, but if the chairman of the Board of Guardians has his way, we shall probably hear more of the matter later on.

### NURSING AT KRUGERSDORP.

An interesting account of her experiences in the Krugersdorp Hospital after the Jameson raid is given by a nurse in *Health and Home* for July 9th and 16th. The writer was one of two nurses who volunteered for the work in reply to an appeal made by the St. John's Ambulance Association in a paper published at Cape Town, where the nurses were residing. High praise is given by the doctors in charge for their consideration for their patients. From a nurse's point of view, the details are very meagre, and we could have wished to have heard a great deal more respecting hospital matters proper.

### AN APPRECIATIVE PATIENT.

"If you please, sir, how can I catch it again?" "Catch what?" asked the dispensary doctor, looking down into the boy's eager face. "Why I thought of course *you'd* know," proceeded the youngster; "I asked mother where I'd best go to catch it again, and she laughed and told me I'd better go to you." The doctor was pressed for time, and he began to get impatient. "Look here, boy," he said, "if you cannot explain what you want you must pass on please, and make way for the next patient." The lad shook his head. "*I* knows right enough what I wants; it's other folks as is stupid! I'm just asking you where I can go to catch the small-pox again. I was discharged from the hospital ship a week back, and I'm just wearying to get back there again!"

### PROGRESS AT GUILDFORD.

We are glad to see that the scheme for the reconstitution of the nursing staff at the Guildford Workhouse Infirmary, with a proposed increase in the number of nurses and in the salaries and improvement in rations, has been carried, in spite of the objection raised by one member of the board in "the interests of the ratepayers' pockets." Fortunately the interests of the patients and the nursing staff won the day.

### HOSPITAL NURSES.

So much nonsense has been written and spoken on the subject of hospital nurses, their treatment, and work, that we have for months been collecting the facts as they exist to-day, and we propose to publish them in a series of articles which will appear consecutively, commencing with next week's issue. In making this preliminary announcement we particularly desire to invite the attention of editors generally to the facts we are about to publish, in order that they may be in a position to keep for easy reference accurate material calculated to cover every point which is likely to arise in regard to this question. There can be no doubt that the best nurses, that is, the real working women of the nursing world, are contented, and that they are healthy in mind and body. This is more than can be said of a great number of young women of all classes who now struggle to obtain admission to the hospitals, though they may be absolutely disqualified by character, constitution, or habit for the calling of a nurse.

### LEISURE HOURS IN THE WORKHOUSE.

Last week the Duchess of Teck opened the annual sale of work at the Paddington Workhouse, in connection with the Brabazon Employment Society. Lady Meath is one of the most active amongst the aristocracy in devising means to alleviate the lot of the poor and afflicted, and this society, which she founded and takes a keen interest in, is one of the most humane of her schemes. There are numbers of old women in the workhouse and workhouse infirmaries who, past active work, find time hang very heavily upon their hands. Lady Meath's visitors provide many of these poor old women with an object and interest in life by providing them with some pleasant task in needlework, the results being disposed of in a sale held once a year. There are 113 workhouses in the metropolitan area, and all have admitted the visitors of the society, who report very favourably of the success of the scheme. In provincial union infirmaries nurses might do much to brighten weary hours if they could secure the help of ladies in the neighbourhood to help in establishing something of the kind.

### SHORT ITEMS.

The Hon. Secretary entertained the members of the Society of Trained Masseuses at tea on the 16th inst. at the Trained Nurses' Club. The distribution of certificates to those who had successfully passed the recent examinations took place afterwards, and the gathering was a pleasant and well-attended function. —Viscount Portman has promised to contribute the sum of £1,000 towards the Extension and Improvement Fund of Queen Charlotte's Lying-in Hospital, of which he is president.—It is pleasant to note that all encouragement is being given to the Nurses' Association in Ceylon. The "Home" for the nurses is now to be enlarged, and three or four rooms added as private wards for the accommodation of patients, towards which scheme two handsome donations of money have already been promised.—The two last Tuesdays, when their annual excursions took place, have been gala days for the nurses of the Bradford Infirmary. Travelling by train to Ilkley, each party, consisting of about twenty-five, drove through the woods to Bolton, where an *al fresco* meal was served, whilst on the return journey tea was in a like manner provided.

Maud 1896 Charles

T.R.H. PRINCESS MAUD AND PRINCE CHARLES.

*By permission, from a photograph by W. & D. Downey, Ebury Street, London, S.W.*

### T.R.H. Prince and Princess Charles of Denmark.

In fulfilment of the wish expressed by the subscribers to the marriage gift to H.R.H. Princess Maud, and of many readers, we have the pleasure to publish to-day portraits and autographs of Our Princess's daughter and husband, who were married on the 22nd instant. It is a common remark, the truth of which is assured, that there is no more united family in the world than that of their Royal Highnesses the Prince and Princess of Wales. Everyone of their children displays the keenest interest in, and most unselfish desire to promote the success of everything with which any brother or sister may be connected at the moment. Of course there are differences of disposition, but there has ever been unity of purpose, and that purpose for each member to promote the happiness of all.

### H.R.H. PRINCESS MAUD.

The Royal bride, who was so happily united to the man of her choice on the 22nd instant, has from childhood been noted for the uniform happiness which has characterised everything connected with herself. In early life, with the exception of measles and whooping-cough, which were to her mere incidents of no importance, she has escaped every childish disorder and has enjoyed the best of health, no matter what her brothers and sisters may have had to contend with in this respect. Princess Maud early displayed unusual ability and a marked taste for art; her drawings exhibit considerable talent, and she has acquired no slight reputation as a painter in colours. Like her mother, she is fond of music, though this art has not appealed to her individually with the same force that it has done to Princess Victoria, who is a considerable musician. Devoted to outdoor exercise and sport, Princess Maud has become an excellent horsewoman, an adept skater, and is quite at home upon the bicycle, which she much enjoys. Accomplished, light-hearted, fall of vivacity, and an excellent conversationalist, Princess Maud is popular amongst her companions and friends, and has enjoyed her life in all probability more than almost any other Princess of her age. By no one will she be more missed than by her sister Princess Victoria. Deeply attached as the two sisters are, there can be no doubt that the separation will be severely felt by both, and we make no doubt that Princess Victoria will again display those attributes of self-denial and cheerfulness which endear her to all, and will make the parting easy for her sister by impressing upon her the feeling that the separation is only to be a brief one, and that it is to be followed by years of frequent and almost continuous intercourse. We can only wish Princess Maud complete happiness, sound health, and freedom from those harassing cares which often oppress the best of men and women. May the future, like the past, have in store for her nothing but happiness, and may every year as it passes bring increased blessing and prosperity to her husband and herself. Princess Maude has shown by her action in refusing to permit any aigrettes to be used in her millinery that she is resolved to exert all her influence to put down the cruelties which are incidental to the decoration of the fashionable world.

### H.R.H. PRINCE CHARLES OF DENMARK.

Prince Charles is a lieutenant in the Danish Navy. Unlike the British Navy, in his country every one who enters this service must commence as a common sailor and work his way up to the rank of officer should he display sufficient talent and capacity to warrant his promotion. Prince Charles has thus acquired habits of discipline and self-reliance which have largely influenced his character and helped to make him what he is in fact—one of the most pleasant and cheerful of companions. Possessed of a fine presence and manly bearing, his thorough mastery of the English language has enabled him, on occasion, to speak with eloquence and force. Prince Charles has ever had the sincerest admiration for Englishmen and England. It is matter for congratulation that the third daughter of our dear Princess of Wales should have the good fortune to be mated to so manly and handsome a man, whose bearing and intelligence have given a favourable impression of his character to all who have had the good fortune to be brought into intimate relations with him. We are confident that the more the English nation come to know him the better they will like him, and that he will eventually become one of the most popular of Royal princes. This view is confirmed by the knowledge that the man in the street has already formed a definite opinion on the quality and character of Prince Charles of Denmark. After the Royal visitors drove away from Drapers' Hall on the 16th July, a discussion arose as to what Prince Charles was. A man in the crowd, addressing the policeman, said: "Yah, bobby, 'e's a sailor laddie, ain't 'e?" and on the policeman replying "Yes, of course," the crowd said "Ah!" with a sigh of relief; "then 'e's all right." The confidence of the crowd is justified by the naval career of Prince Charles. He has won his way in the Danish Navy by sheer hard work, and he is a devoted student of naval tactics and has an intimate knowledge of most books published on this subject and on seamanship. When selected recently for special service as commander of the "Heimdal," he displayed so much energy and success that the inhabitants of Iceland entertained him at a special banquet at Reykjavik in commemoration of his services.

The nurses who had the good fortune to be present at Marlborough House when the marriage gift was presented to Princess Maud and Prince Charles will never forget the gracious words spoken by him on that occasion or the evident sincerity and ease with which they were uttered. It is pleasant to be able to state that among all the presents which have been received there has been none which has given greater satisfaction or been more generally admired than the gift of the Pension Fund nurses, which constituted a spontaneous expression of the deep regard which every nurse in the British Empire feels for their Royal Highnesses the Prince and Princess of Wales.

### The Royal Wedding.

"What did the bride wear?" is one of the first questions asked when a wedding is under discussion, and therefore we hasten to mention the dress in which Princess Maud of Wales was attired for her marriage on July 22nd.

Of course, it was a pretty robe, and graceful withal, for the Princess of Wales and her daughters always avoid extremes of fashion, and set examples of moderation and good taste. Princess Maud wore a beautiful white satin dress, not very full in the skirt, and trimmed with a simple ruche of white chiffon round the hem, sprays of orange blossom, myrtle, and jasmine, being laid amongst the soft folds. The satin itself was woven in Spitalfields, and the bodice, trimmed with chiffon and sprays of flowers like those on the skirt, was finished at the waist with a belt of diamonds set in silver. The marriage was celebrated in the private chapel at Buckingham Palace, and the road from Marlborough House to the Palace was lined with Coldstream and Scots Guards, and very imposing they looked.

The bridegroom, Prince Christian Frederic Charles George Valdemar Ascel, is very tall; he is an officer in the Danish Navy.

Princess Maud, whose full title is Maud Charlotte Mary Victoria, looked a very dainty little bride. The wedding ring was sent from Wales, and presented at Marlborough House by a deputation of 330 persons on Tuesday last, an address in Welsh being read, and in addition to appropriate congratulations conveyed in it, a graceful tribute to Denmark was paid in the words, "That august home to which we are already beholden for that gracious lady whom we venture to look upon as our own Princess." The ring was enclosed in a very fine casket.

The presents were costly and numerous, and were arriving up to the last moment. Many were previously on view in the large dining-room, and those too large to be shown, such as carriages and furniture, were represented by photographs.

Her Majesty the Queen gave a magnificent necklace of diamonds and rubies, silk and satin brocades for dresses, and some very rare lace.

The gifts of the parents of the bride and bridegroom were particularly handsome ones, and many other treasures would require a whole "Nursing Mirror" to be exclusively devoted to their reflection.

The elegant tea service and table presented by the Pension Fund nurses received a good deal of notice from the numerous visitors who were privileged to view the costly exhibition of wedding gifts, and our readers will not forget Prince Charles' kindly promise on its presentation to the effect that he and his bride in using the service would have pleasant thoughts of the givers. It is flattering to the donors that this present was chosen as one of a dozen others of which illustrations were given in the Special Wedding Number of the *Daily Graphic.*

The Queen came up from Windsor on Tuesday and proceeded to Buckingham Palace, where she remained till Wednesday evening. The bridesmaids were Princess Victoria of Wales, Princess Victoria of Schleswig-Holstein, Princesses Ingeborg and Thyra of Denmark, Princesses Margaret and Patricia of Connaught, and Lady Alexandra Duff (daughter of the Duke and Duchess of Fife).

### In the Streets.

The gleam of the white tents in the grounds of Marlborough House reminded more than one Pension Fund nurse of the hospitality which had been received on the same spot last year. But on Wednesday the garden entrance which had been opened for the admission of the nurses remained closed, all the company issuing from and returning to the State entrance.

Before the first procession passed down the narrow street leading to St. James's Park and Buckingham Palace, the pavement was packed with an enthusiastic and orderly crowd. Suddenly someone looked up at the high wall to the left of the garden entrance, "What a dear baby!" she cried; "now who can that be?" Many were the conjectures that followed from the lookers-on. One voice in the crowd joined in the discussion, saying: "'Taint likely as they'd let a prince sit up on a wall! Now just you consider! Would 'is ma or 'is gran'ma allow such a thing?" "Why, a most respectable elderly gent brought a red cushion just now, and the baby's a settin' on it at this very minute. An' 'is nurse is holdin' 'im most careful," said another. "He's dressed most sensible. Clothes as white as snow, and goes to the wash regular I should say. Neat 'at too. Looks like a leghorn and a full border like a cap front, same as the Queen (God bless her!) wore when she was a kid," remarked someone else. And then the baby was forgotten as "Here they come!" passed along the pavement. The bridegroom, stately and composed, with his brothers from Denmark, occupied one of the gorgeous state carriages. The moderate pace at which these vehicles proceeded was fully appreciated by the spectators. Whilst every occupant was scanned critically, the Princess of Wales was cheered with enthusiastic and prolonged energy. She turned her jewel-crowned head for an instant to smile at a little group of scarlet-cloaked children, whose eyes were admiringly fixed on "that beautiful lady." By her side Princess Victoria of Wales was seated, and she, too, smiled and bowed at the loyal throng of onlookers. An equally cordial greeting awaited the Prince of Wales, who was himself radiant with fatherly pride in the dainty little bride on his right hand. Her head was a little bowed forward, and the fair face was slightly flushed as she was driven slowly past the closely-packed spectators. "A bonnie bride." "*That's* worth seeing." "Now I'm going; I've seen all I want. Nothing will beat *that*," were the various exclamations as the units which composed the throng began to disentangle themselves. Some neat nurses hurried off. "I must get back," said one. "Yes, and hasn't everybody been nice and good-tempered? I shall have a great deal to tell the patients this evening; they love to hear about the Royal Family." "What was 'Our Princess's' gown made of?" "I'm sure I don't know. I couldn't see anything beyond her sweet face and the flash of the jewels." And presently the pealing bells told the old, old story of another pair of lovers made "man and wife."

When the Royal bride and bridegroom drove away after the wedding, the presence of mind of Princess Maud was much admired. On entering the carriage she prepared for a siege of rice by screening herself with her parasol. Poor Prince Charles, who had no similar protection, had to bear the brunt of a severe onslaught.

As an instance of the solicitude the Queen always shows to the suffering, we may mention a little incident which occurred soon after Her Majesty had taken up her position at the windows of the Yellow Drawing-room at Buckingham Palace to witness the departure of the bridal party. A lady was brought in a fainting condition to the party of the St. John Ambulance Brigade who were stationed outside the gates of the Palace, and had to be placed on a stretcher and conveyed inside the gates of the Palace to be attended to. From the window at which she was sitting Her Majesty had a full view of the occurrence, and sent out no less than three messengers to enquire as to the patient's condition. The last of these was an Indian official who was in attendance on Her Majesty in the room where she sat, and he did not return until he was satisfied that the lady was better.

### Hygiene: For Nurses.

By John Glaister, M.D., F.F.P.S.G., D.P.H.Camb., Professor of Forensic Medicine and Public Health, St. Mungo's College, Glasgow, &c.

### XVI.—DETECTION OF IMPURITES IN WATER. MODES OF PURIFICATION OF WATER.

The impurities of a water may consist either of (1) suspended matter, or (2) organic matter, or (3) mineral substances derived from organic sources, in solution, which are removable by adequate filtration, the former entirely, the latter to a varying degree. Let us meanwhile deal with the impurities dissolved in the water. Their detection falls within the domain of the chemist; but without trenching unduly in his field, it may be pardonable if intelligent persons should desire a little knowledge of the subject—sufficient, at least, to enable them to sound a note of alarm. As chemical solutions nowadays can be purchased ready made for testing, the mere operation of testing becomes mechanical; the difficulty emerging at the interpretation of the results of the tests. The impurities to be looked for are those which are animal in origin, and may be taken, when found in amount, as evidence of sewage contamination. They are liable to be present in waters from surface-wells, owing to soakage of excrementitious fluids. There are two substances which are not difficult to detect, which, too, when found in notable quantity, in waters from certain sources, point indubitably to animal pollution. These are chlorides and ammonia. Urine is their common source. Sodium chloride—common salt—is always abundant in healthy urine. So is urea, which, when acted upon by a microbe, is decomposed into salts of ammonia. Of themselves they are not harmful, but owing to their common association with more potent factors for ill they may be taken as danger signals.

Chlorides may be detected by adding to a test tube half-full of the water to be examined a few drops of a dilute solution of nitrate of silver (lunar caustic) and a like amount of dilute nitric acid. A white haze, or turbidity, or precipitate indicates chlorides, and the denser the haze the larger the amount of chlorides. A marked haze indicates a suspicious amount.

Ammonia is detected by adding in a similar tube a few drops of Nessler's solution. If ammonia be present a yellowish or yellowish-red colour will develop, the colour deepening in intensity with the amount of ammonia present. A marked yellow colour indicates danger. Each of these reactions is best seen by looking down through the column of water in the test tube; in the first, against a dark ground, and in the second against a white surface, and by placing alongside a second test tube, filled with water to the same level, but to which the chemicals have not been added. The slightest difference in colour may then be detected.

The presence of organic matter points to contamination from animal sources if the chlorides and ammonia are present in notable quantities. Organic matter is detected by a weak solution of Condy's fluid, made by adding two drops of Condy to half a teacupful of pure water. Of this add four drops to a test tube containing water, with one drop of dilute sulphuric acid, and warm contents of tube. The pink colour disappears if organic matter be present.

Purification of Water.—Those impurities of water due to sewage contamination which are most dangerous to life are rendered innocuous by the simple operation of boiling the water before it is used. To make it palatable for drinking it should, after cooling, be re-aerated as follows: Pour the water forcibly from one vessel to another, in the open air, or by means of the rose of a watering-can, which by dividing the liquid into fine streams causes it more easily to reabsorb atmospheric gases. It should then be kept covered in a cool place, and, in summer, preferably in a porous earthenware jar.

Suspended matters, such as mineral matters, particles of floating substances, or even animate beings such as water insects, or, upon rare occasions, small fishes might find their way into the main supply pipes unless the water be strained or filtered at its source. The water of Loch Katrine is not filtered before distribution, and curious tenants are sometimes found in the water-pipes. Our neighbour experienced one a few years ago. The supply to the kitchen tap was suddenly and completely cut off, although it was as usual in the other parts of the house. On opening the pipe it was found that the cause of the obstruction was a small trout measuring about four and a'half inches in length. It is difficult to say whether this fish made a subterranean voyage of thirty or seven miles, the respective distances of the loch and reservoir.

Where suspended matters are present, filtration must be resorted to by the water companies. Filters are constructed on the grand scale, and the filtering materials used are sand, small gravel, large gravel, shells, and small boulders, in that order from above downwards, the respective thicknesses of the layers of each varying according to the opinion of the engineer. These filter-beds, in addition to straining suspended matter, remove to a considerable extent both organic matter and microbes. This is purification on the large scale.

Fig. 29.—Chamberland Filter. Fig. 30.—Porous "Bougies."

Household filters are the means adopted to purify on the small scale. Much has been written lately condemnatory of these filters in respect of their failure to achieve what is expected of them. They fail chiefly in not arresting microbes, and in respect that the filtering media are not cleansed often enough they permit of the multiplication of such microbes—in short, become breeding grounds for these organisms. On the other hand, if the filtering media be repurified at regular short intervals their action is quite safe. The media employed are: (1) Carbon, animal, vegetable, or mineral, sand in powder or in block, iron, in some form, and asbestos. To purify the first and last named it is only necessary to subject them to the influence of fire, to a red heat, and the others by washing several times with boiling water. The only filters which do not permit the passage of microbes are the Pasteur-Chamberland and Berkefeldt filters, the former of which is made of tubes of unglazed porcelain, and the latter of tubes of compressed diatomaceous earth, spoken of as "bougies." Fig. 29 shows the Chamberland filter fixed in position on the water-tap, and Fig. 30 shows the porous "bougies" through which the water filters.

Distillation is another mode of purifying water, and is now commonly adopted in ocean-going steamers for the supply of potable water from seawater. This process insures purity, and, after re-aeration, no better water can be had. It is nature's mode of supplying the earth with water.

Fig. 31 illustrates the apparatus for distilling. On the left of the figure is the vessel containing the heat, on top of which is the retort containing the fluid to be distilled; this

Fig. 31.—The Still and the Worm.

opens into the condensing apparatus, a tube or "worm" lying in a vessel containing cold water. The circular dotted parallel lines indicate the "worm," which presents, by its spiral form, a larger condensing surface. The distilled fluid escapes at the bottom of the condenser by the small tube.

To rid water of hardness-producing mineral substances—chiefly lime and magnesia—is of importance where it is necessary that a soft water only should be used, as in certain commercial operations. This may also be deemed a form of purification, inasmuch as it purifies it of materials which, for the purpose intended, would be objectionable. But this purification is one of expediency more than necessity.

### Colonial Nursing Association.

### A DRAWING-ROOM MEETING.

The Hon. Lord Loch, G.C.B., G.C.M.G., presided at the meeting held, by kind permission of Mr. Cuthbert Quilter, M.P., at 74, South Audley Street, on the 15th inst.

Amongst those present were Lady Lucy Hicks-Beach, Sir John Goldie, Governor of Trinadad, Sir W. Maxwell, Governor of the Gold Coast, Lord Stanmore, Dr. Gage Brown, medical adviser to the Colonial Office, Mrs. Charles Robinson, Mrs. Charles Hobhouse, Miss Rosalind Paget, Sir Hubert Jermingham, Mrs. Francis Piggott, hon. secretary, and others.

Lord Loch, addressing the meeting, said he considered the chief object of the present meeting was "to announce themselves" to the public. The Association aimed at providing trained nurses for Crown colonies, and this had the approval of the Secretary of State and many others. Mr. Chamberlain had issued a circular letter to all the Crown colonies. They had no intention of relieving the colonies of their own responsibilities, they desired only to organise a supply of qualified nurses for the English communities abroad. One nurse had worked in the Mauritius with marked success, and the Association has now sent out to the same colony nurses Boltnoell and Cunnington, whose services would be most acceptable there. In colonies where societies already existed for supplying trained nursing to the English, they could either continue their work independently, or become associated with the new society. Of course, money would be required for initial expenses to pay for travelling and maintenance of nurses, &c., but it was expected that for this a very few hundreds would suffice, as the English residents would gladly pay the fees of the private nurses when they had the benefit of their services.

Dr. Gage Brown said that all the great things done in the world had been originally started by some one person, whose energy and interest attracted others. Miss Nightingale had initiated skilled nursing in England, and Mrs. Piggott had formed a scheme for securing equally good nursing for the colonies. He thought that if thoroughly trained and competent nurses were sent out, they might train the local women to be of more use.

Sir William Maxwell said he could speak as one of those who would gladly avail themselves of the services of English nurses for their own people. He had seen the good work done abroad by both Roman Catholics and Protestants in hospitals, and on his return to the Gold Coast he should certainly benefit by the movement now inaugurated, but of course there would be difficulties at first in carrying out this scheme.

Sir John Goldie also foresaw difficulties of administration.

Lord Loch thought they might for the present steer clear of the question of discipline, which would no doubt be satisfactorily settled. The subscription list at present showed between £300 and £400.

The Government Secretary of British Guiana said he had on former occasions had to do with importing nurses, and they would, certainly, in this case be under the authority of this association only when nursing private cases. If they entered hospitals they would be under and protected by the local government. He felt sure the nurses would be able to pay their own expenses and up-keep if the initial costs were met. It would be the work of the association to supply, by co-operation with hospitals and institutions in England, such nurses as the colonies might require.

Mrs. Piggott said that she did not know that the duties of an hon. secretary would include speaking. She had found the want of nurses in Japan, and in the Mauritius young Englishmen died of typhoid, and had no proper nursing at all. She knew of four fatal cases on one estate last year. Many lives were lost for want of competent nurses. The sympathy of friends at home must certainly be with their sick friends abroad.

A vote of thanks to Lord Loch for presiding, and to Mr. Cuthbert Quilter for the use of his house, concluded the meeting.

### Minor Appointments.

Hahnemann Hospital, Liverpool.—Miss Florence Lea has been elected a Sister at the above institution. She was trained at the City Infirmary, Birmingham, and holds the L.O.S. certificate. She held the post of sister in the children's ward, and later in the women's surgical ward at the City Infirmary, and subsequently took up private nursing on the staff of the nursing institution at Newcastle-on-Tyne, where she remained for two years and three months.

Swansea General Hospital.—Miss Agnes Sherring has been appointed Night Superintendent of the above hospital. She was trained at the Royal Southern Hospital, Liverpool, and held appointments as nurse at the Southport Infirmary for six months, at the South-Eastern Fever Hospital, London, for four months, at the Southampton Infirmary for two years, and the Liverpool Stanley Hospital for three years. We have received copies of Miss Sherring's testimonials, which are most excellent.

Eastern Hospital, Homerton.—Miss Annie Ellershaw, who was trained at the Sunderland General Infirmary, and Miss Isabel Reynolds, who was trained at the Wolverhampton and Staffordshire General Hospital, have been appointed Charge Nurses at the Eastern Fever Hospital.

Basford Isolation Hospital.—Miss Pringle, the matron, informs us that she held the post of sister at the Stillorgan Convalescent Home, Dublin, for one year and a half, and not one year, as was stated.

### A Book and its Story.

### "A DARN ON A BLUE STOCKING."

There is a certain attractiveness in this little story* which lies outside its literary merits. The writer's name, by the way, is new to us, and it may be urged she is not possessed of any very matured experiences as a novelist. G. G. Chatterton betrays her sex by the importance she attaches to trifles, and in various minor qualifications (or disqualifications) which distinguish feminine writing; but "A Darn on a Blue Stocking" has its own charm, and the authoress certainly possesses the gift of telling a story in a simple and a delightful manner.

It is the history of a modern young woman—the so-called "Blue Stocking"—who, after all, is not a very modern young woman, by name Valencia Arbuthnot, to whom we are introduced on the eve of her career, which is a self-imposed one.

At first sight Valencia's circumstances do not appeal to one's commiseration, as she is luxuriously housed with her brother, and is possessed of a comfortable income of her own. But the girl is fired with the ambition to distinguish herself in spheres far removed from the quiet seclusion of the ancestral home, and desires she interprets to her brother. "The whole affair, Tom dear," she explains to him, "lies really in a nutshell. Here am I left, as you may say, upon your hands, and possessed of but £300 per annum. I hate being on any one's hands, and I hate £300 a year. My scheme, therefore, is to endeavour to work and make money—and why should you try to gainsay me in my attempt?"

The brother suggests a practical way out of the difficulty. "Why you will not marry Edward Thornton is more than I am able to conceive; a good fellow, well born, and rich, one whom you have known all your life, and whom you really must like," Valencia's brother argued. But the girl's mind is made up. Tom's arguments are of no avail; she had decided, when she refused the desirable suitor, that a life of independence was the proper life for her—she will take up art as a profession. Soon after this Sir Thomas takes to himself a wife, and Valencia has further motives for putting her intentions into practice, and she leaves the country home with its lawns and its quiet terraces. She had had enough of it, she assures herself, enough of the irresponsible, dawdling, do-nothing routine of the old existence; and she wanted the stronger current to breast—"the pulsing life of the great city, and its throb, and ebb and flow of humanity; and her restless spirit told her that nature may be beautiful but that art is satisfying."

Valencia Arbuthnot's new quarters are humble ones in an unobtrusive street in Kensington. In the same house, on the floor below her, it happened there lodged a Mr. O'Brien, round whom, as the story goes on, a certain interest is attached. He is described as a hard-working journalist, a man with a future before him. As a natural consequence, and through a strange interposition of Providence, the two meet—become acquainted. The girl makes an impression there and then, we are told, upon the man's impressionable Hibernian nature.

Their first meeting was only introductory of many, and the acquaintance, formed on an accident, ripens into friendship. Both have their work, so it is only in certain off hours that there is leisure for the interchange of thought, growing dear to each. But neither Valencia Arbuthnot nor her fellow lodger knew whither this friendship was drifting; they were content to let it drift, unheedingly, thoughtlessly. They had so much in common, these two—it all fell in so naturally, that they should express an interest in each other's labours. Her painting was not an unmixed success to the woman, nor was his writing to the man, and a mutual bond seemed round them such as comes to comrades.

Valencia's new career suited her well, and she carried out all her determinations of making her life a serious one in its purpose. She worked hard, and spent her income principally upon her art education, little of it upon the ordinary etceteras of everyday existence; and whilst she was occupied and energetic in her present, so she was sanguine as to her future.

And after a while orders came for her. These portraits, and her paintings, she showed to Mr. O'Brien "in the extra little room that was part of her domain, and which she had converted into a sort of little studio," and he criticised them admiringly—how could he have found it in his heart to do otherwise in sight of the bright eager face turned expectantly to him? And, besides, her paintings really were clever, and above the limits of the ordinary amateur endeavour. And he, seeing what interest she took in such things, brought up to her MSS. of his, just to show her what an immensity of large papers, closely written over, were necessary to finish even a short story or essay.

Though it was a work-a-day world in which the two passed their separate existences, yet it had its Arcadian lulls, too. Little happy intervals, when spring hours were spent together, in picture galleries, or in Kensington Gardens among the glories of the coming summer.

Thus the affair "marched quick," as the French say. A little cloud of Miss Arbuthnot's horizon presented itself in the shape of a pathetic little typewriter, engaged by Mr. O'Brien, as greater demands were made upon his pen. Somehow, whether it was coming events forecasting their shadows, or a certainly womanly intuition on her part, Valencia began to regard the journalist's co-worker with a certain jealousy. But the girl never told herself she was jealous; she was as unconscious of the exact nature of her feeling towards the little typewriter, as she was up to now of the real nature of the regard she bore to the employer.

At last Valencia discovers that her feelings for the fellow lodger are less of a platonic nature than she would wish, and the discovery was further humiliating to the girl's proud, independent nature when it came to her, together with a great uncertainty as to the man's feelings towards herself. Then followed little misunderstandings, and the two drift slowly apart—not to meet again in the old happy manner; and when they do meet again, after several years, it is unexpectedly one night at a London assembly.

And it is no longer as the toiling artist of humble circumstances that Valencia accosts once more her comrade of the former days, but she stands before him in be-diamonded splendour as Edward Thornton's wife.

And Mr. O'Brien is not alone; his wife accompanies him—the pathetic little typewriter, for whom Valencia had once been sorry, and later on jealous, and who would never know she had become her employer's wife through a misunderstanding only with the woman her husband had loved in the old lodging-house days.

And so it is that the man and the woman meet again who were so much to each other.

"Face to face they looked at one another, and in some occult fashion learnt that in those old days they had returned each other's love. All the bitterness on one side, the sorrow on the other, of misconception swept away. For a moment hand clasped hand in silent revelation—comprehension.

"But it was a farewell, as well as birth; a burial. . . . Both knew that this was so; that they intended to make it so. Valencia's eyes turned very soft and dewy. Then she drew away her hand, and, turning, looked out again upon the world's company which they must rejoin, and 'We had better go back,' she said, in a voice of gentle softness."

And then they went back to the separate calls life had for each, with an understanding of the past which, if it had its sorrow, possessed its happiness, too.

* "A Darn on a Blue Stocking." G. G. Chatterton. London: Bellairs and Co., 1896.

### The Unemployment of Atkins.

By a Superintendent of the Indian Nursing Service.

Referring to *Truth* of April 30th, I was reminded by the above heading of one of the articles of a subject I have thought much about during late years, and one that perhaps some day may be carried out. That is, the employment of old Indian nursing orderlies of military hospitals as male nurses in England.

How often we hear of men objecting to be nursed by women, and how unsuitable it is for many women nurses to be sent to nurse men in chambers and in colleges. There are many diseases that men are liable to which are utterly unsuitable that women, and in many cases young women, should be sent to attend. As an old sister of men's wards, I know. I could mention twenty diseases, but I will take one, a very common one—that of paralysis; how much more suitable in utterly helpless cases of that kind it is that a man should look after a man, where he is unable in any way to assist himself. There are matters and duties in connection with such cases, where it is hard to say which it most painful—for the patient who has to be attended to or the nurse who has to attend him.

Every year there are soldiers returning from India, time-expired men, who have been employed in the station hospitals as nursing orderlies. Some of these men are excellent nurses, holding both ambulance and nursing certificates. Some of them have had, off and on, four or five years' experience in nursing serious cases, such as pneumonia, enteric fever, abscess of the liver, &c.

These men are trained by the nursing sisters (themselves trained women), in all the duties of nursing, such as lifting, bathing, sponging, feeding, wet packing, bed-making, bed shifting; temperature, pulse, and respiration taking; giving of medicines and stimulants; making notes and observations of the cases; writing reports; preparing food, such as Benger's food; diluting milk, preparing beef-tea, chicken broth; peptonising milk; making whey, junket, &c. They are also taught how to apply dressings, making and applying poultices and fomentations, &c., and looking after the patients' backs, and everything else that ministers to their comfort, and how to act in emergencies.

Why should all this training be thrown away and wasted? Why should not a corps or association be found for the employment of these men? Taking, of course, those only who hold good testimonials from the hospitals they have worked in, and have from their regiments good conduct certificates. Ambulance certificates alone I should not consider sufficient. If such a society was started I am sure it would be appreciated.

Men who are in the true sense of the word nurses are excellent nurses. I have trained both women and men, and superintended both, and I know how good many of these soldier nurses are. Take those who have been officers' servants, as well as having trained under the Indian Nursing Service, and you will find that if you put them down in a bachelor quarter to nurse a sick man, they would be doubly as useful as the ordinary trained private woman nurse that is going about. They would do the house work, and would have more strength for lifting, &c. The fact of their nursing duties being voluntary and not compulsory has given us, as a rule, men of a natural aptitude for the work. My experience has been that a loafer, an intemperate man, a lazy one, one who slept on night duty, one who was unkind, one who was physically or mentally incapable of the work, very quickly was returned to regimental duties.

I have worked side by side with men who would have done infinite credit to the training of some of our highest recognised training institutions in England. I have seen their powers of endurance through the hottest days in summer, in one of the hottest stations of the plains; I have seen their gentle care of the dying; I have seen their reverent treatment of the dead; I have seen these men, whom the world has never properly appreciated, after a hard night's work and a broken morning's sleep in a crowded orderlies' bungalow, get up and make a wreath or a cross of flowers to place on his dead patient's coffin, or perhaps under a scorching afternoon's sun, with his old Khaki helmet jammed down on his head, searching the dusty garden hedges to find some green to eke out his flowers which he has probably had to buy or has walked far to find.

The fact of their military discipline and training makes them able to intelligently carry out orders; give them written directions and time-tables, and, as a rule, you find them carried out to the letter.

Should such an association be started privately, or by Government, it would be as well for the men to put their names down for it six months before leaving the country—I mean India—and then, perhaps, Government might arrange for them that they should be especially instructed by the Subordinate Medical Staff in those duties which would be most essential for a male nurse as a male attendant to know.

### Royal British Nurses' Association.

The annual meeting of the Royal British Nurses' Association was held on the 22nd inst. in the Great Hall, St. Bartholomew's Hospital. Sir James Crichton Browne, in the absence—owing to the Royal marriage—of the president, H.R.H. Princess Christian, occupied the chair. The proceedings throughout were of a most stormy character.

The treasurer presented his financial statement for the year ending March 31st, 1896, showing receipts on the general account amounting to £1,219, including a balance brought forward of £317, and expenditure amounting to £1,122, leaving a credit balance of £97.

The balance-sheet gave rise to much acrimonious discussion, in the course of which Dr. Bedford Fenwick said that the association was now on the verge of bankruptcy, a statement warmly combatted by the Treasurer and the Chairman.

The annual report presented by the Medical Honorary Secretary also gave rise to a scene, Dr. Bedford Fenwick objecting to its being presented and adopted on the ground that it was not the report of the Executive Committee, and, therefore, not sanctioned by the bye-laws of the association. The report was, however, adopted by a large majority.

After another scene over the election of the General Council,

The resolution which we referred to last week (of which notice had been given by Miss Margaret Breay) expressing strong disapproval of the methods of management pursued by the present Executive Committee, was brought forward. It was at once ruled out of order by the Chairman, on the ground that notice had not been properly given, the resolution not having been sent in a registered letter.

Votes of thanks to various officials concluded the proceedings, but even these were not allowed to pass without objection.

### The Treasurer's At Home.

The Hon. Treasurer and Mrs. Langton gave an At Home at 62, Harley Street after the annual meeting. Many distinguished guests were present, in addition to a large gathering of nurses, and everybody enjoyed the excellent music, recitations, and other good things provided for their entertainment. The heartiness and good-fellowship of this At Home were good to see, and bore excellent testimony to the popularity of the officers and council of the association and their widespread influence.

### Where to Go.

East London Exhibition, People's Palace, Mile End Road.—Miss Gethen (formerly Sister Queen of the London Hospital) will relate some original stories on "Ward Pictures of Hospital Life" at five o'clock and seven on Thursday, July 30th, and at the same hours on Thursdays in August.

### Nursing Arrangements at Clapham.

THE nursing staff at the Clapham and Wandsworth Infirmary do not seem to appreciate the recent attempt to hold them up as objects of pity. "We have never been paragraphed in the papers before," said one of the workers, "and we don't like it!" One or two guardians are much exercised in their minds because the nurses leave, and they fail to see that it is the natural sequence of a three years' course of training. We are informed that nurses who have left the infirmary to take other posts have filled them with distinction to themselves and credit to their training school. There is no doubt that the nurses' sleeping quarters are totally inadequate for the present staff, the cubicles being inconveniently small; but the mess-room is good, and the recreation-room very pleasant. During the summer for about two months, it is, unfortunately, necessary to devote this room to the service of the patients, whilst the wards undergo cleaning in turn. We are under the impression that the Local Government Board does not by any means approve of the present dormitories, and the sooner the guardians make up their minds to build a proper nurses' home the better. Great improvements have been made in the last few years, and the medical superintendent gives lectures to the probationers, and an annual examination is held. Fire drill is also taught to the nurses, who are said to be expert at it. They have a small library of their own, and other advantages. On their monthly "day off," they do not go on duty at all. They have also a half-day off once a month, and certainly it would be difficult to find a healthier and nicer-looking set of nurses than the present staff. If the guardians who think the probationers improperly lodged will be good enough to practically advance the desired new home, they will earn more gratitude than they are likely to secure in their present action. They might also include by their reforms an extension of the annual holidays. Pending such changes, it is pleasant to find so many good workers intent on making the best of existing conditions and content to include in their daily work many small duties which only the newest of probationers miscalls "menial." The matron and her faithful staff of sisters and nurses prefer to speak of these necessary homely labours as "manual" ones. A terrible compound fracture (doing well) and many serious medical cases show that there is no scarcity of acute cases at the infirmary, which is so pleasantly placed on St. John's Hill. The majority of the guardians have taken much interest in the establishment and maintenance of a creditable nursing staff that will no doubt succeed in convincing the present little band of agitators that it rests with themselves alone to bring their nurses' quarters up to date. The matron seems for many years to have done her best to raise the nursing, a task in which she has had the warm support and encouragement of the medical superintendent.

### Everybody's Opinion.

[Correspondence on all subjects is invited, but we cannot in any way be responsible for the opinions expressed by our correspondents. No communications can be entertained if the name and address of the correspondent is not given, or unless one side of the paper only be written on.]

### THE CLAPHAM AND WANDSWORTH INFIRMARY COMPLAINTS.

THE NURSES of CLAPHAM and WANDSWORTH INFIRMARY write: We saw your comment upon the scandalous reports which have been spread about our infirmary in last week's HOSPITAL, and knowing that you would wish to be just and fair, we think it only right to tell you the other side of the picture. There is even a worse report than the one you alluded to, in which it is stated that the work of the nurses is that of labouring men, that many go without their food because it is a scramble, and that one nurse is compelled to sleep in a cellar, and others have to dress in the corridors. We (the nurses and sisters) were all so indignant at this, that we at once addressed a letter to the editor of the paper in which we first saw it, but to our disgust it has never been inserted, which we consider a most unjust thing. Why should any paper scatter a reported scandal all over the country, and then refuse to print the explanation or contradiction? We, the nurses of the above institution, appeal to you for justice. Will you kindly publish this or such extracts of the following facts as you think suitable? (1) Hard work: Most of your readers will know that nursing is always hard, especially to the novice, who usually has some airy notion that nursing consists of "shaking up pillows," as is often depicted in novels. We were not deceived about the work, but were before commencing it made to understand what to expect. Under these circumstances we think that no one is justified in complaining, or if they think they have a grievance they should be fair and square and report it in a straightforward way. There has been an increase of staff more than once, and as the work is exactly the same, how can it be harder now? (2) Badly paid: I think you will find that the pay here is equal to that at other infirmaries. (3) About the scramble at dinner: If some are rude enough to scramble, of course it makes it unpleasant. If any nurse goes without her dinner it is entirely her own fault, for the food is good, the supply ample, and all are allowed one hour for the meal. (4) Improperly housed: Inconvenience in the sleeping accommodation is caused chiefly by the increase of staff. Most of the assistant nurses sleep in cubicles, partitioned off with curtains at the end of each cubicle in a large dormitory with a passage down the centre. One has a smaller cubicle than the rest, so comes outside the curtain to dress if she likes; the other assistant nurses share rooms, containing two or three beds, as the case may be. The nurses and sisters have nicely furnished single rooms. The number of resignations in fourteen months is thirty-one, not forty, as stated. This includes those who came one day and left the next or in a few days, not having given the place a fair trial. Most of the other nurses have gained their certificate, and left to take up different branches of nursing, fever, district, private, &c., and are doing well. Some have also left to be married.

### NURSING IN SOUTH AFRICA.

"A NURSE" writes: I read in a paper that Lord Loch, speaking at the Imperial Institute, said there was a good opening in Africa for women as trained nurses, typists, shorthand writers, and telegraphists. As an English-trained nurse who has worked some years in South Africa, I beg to differ from Lord Loch's statement as to there being a good opening for trained nurses. There is a limited demand for English-trained nurses, but they must be above the average, good, strong, healthy, sensible women who would be able and willing to turn their hands to anything, not afraid to clean their patient's room or cook their food. Life here is very rough to people accustomed to the comforts of England; in fact, an Englishwoman cannot realise the difference, or how much she is waited upon in the old country, until she comes out here to find a lack of comfort and convenience on every hand. The salaries here are much higher, it is true, but then clothing and everything else are so much more expensive than at home there is very little left to the good at the end of the year. I should not advise nurses to come out unless they have private means or friends in the colony to fall back upon. Then they must make up their minds to make the best of a rough life; many English nurses grumble very much at the hardships and discomfort of colonial life. Our principal hospital, the New Somerset, Cape Town, trains its own nurses, and prefers colonial women, though there are a few English on the staff. Kimberley Hospital takes English-trained nurses, also trains; there are many small hospitals, but generally their wants are met by the colonial-trained nurse—in fact, our main want in South Africa is good female servants. The best way is for English nurses to have their names put on the Register of Trained Nurses in the colony, for which they will very possibly have to undergo an examination, unless their certificates are very explicit as to the quality of training she has received. There is no fee for registration; and the offices are at the Parliament House, Cape Town.

### For Reading to the Sick.

### AN OBJECT IN LIFE.

**Motto.**

It is not enough *not* to do; you are bound to act.

—*Mazzini.*

**Verses.**

What is our work, when God a blessing would impart?
To bring the empty vessel of a needy heart.
To see the face of God, this makes the joy of Heaven;
The purer then the eye, the more joy will be given.
God loves to work in wax, not marble—let Him find,
When He would mould thy heart material to His mind.
Wouldst thou abolish quite, strongholds of self and sin,
Fear can but make the breach of love to wander in.

—*R. C. Trench.*

The flower is but a little thing,
It perfumes all the gales of spring;
God feeds it with His dewdrops bright;
And never yet the heart has beat,
Too mean, too lonely, too unmeet,
To do its proper part aright;
Nor hand has been too weak or small,
To work for Him, Who works for all.

—"*Heavenly Thoughts.*"

I built my nest high up and free,
Thou, with Thy wind did'st shake the tree,
Telling them nought was safe beneath the stars;
But when I set Thee all at nought,
I fell to caverns of dark thought,
When all around me seemed night's everlasting bars.

Nor will I strive Thy love to gain,
Which none can strive to win in vain,
But still some hand unseen doth hold me back;
Then I for that will strive the more,
That I may learn that I am poor;
Our poverty to know is all the wealth we lack.

—*Isaac Williams.*

Our duty down here is to do, not to know;
Live as though life were earnest, and life will be so.

—*Lytton.*

**Reading.**

"Is any among you afflicted? Let him pray."—*James* v. 13.

Illness is not a cessation of life, but a different life, which has its own ideals, its own duties, its own opportunities, even its own amusements. It is a chapter of your life, which has just as much meaning in it as any other when it closes, by death or by recovery; you may be able to see that it had even a deeper meaning—that is, if you live it "with intention," not if you slip through anyhow, from day to day Perhaps you know how detrimental this "hand to mouth" method has been to your past chapter of active life. You may have seemed to other people a busy and even methodical worker, one who used life to the full, and yet, in your heart, you may know that you never had the energy and courage to "begin to live"—your private habits of devotion were always going to be set straight some day—a day which never came—your duties were never marshalled and co-ordinated, so that all had their fair share.

If you were sufficiently self-disciplined to have lived a life of proportion in health, you will instinctively do it in ill-health; but, if you did not, begin now that you have had to start a new chapter. Your life, even before, was full of holiness—let it now have the beauty of holiness, which is impossible without that interior order, the absence of which was definitely known only to you, but indefinitely felt by all around you. Arrange your life, on all days when it is possible; keep to your plans; when extra pain or weakness interferes with them, accept it as God's own rearrangement, not as a disturbance to be fretful over, or as a reason for losing heart in your efforts at regularity.—*L. H. M. Soulsby.*

### Queen Victoria's Jubilee Institute for Nurses.

We are asked to state that two umbrellas and an apron remain unclaimed at St. Katharine's Royal Hospital, Gloucester Gate, Regent's Park, N.W. They will be sent to the owners on application to "The Inspector" at the above address.

### Appointments.

Turriff Hospital.—Miss Annie Tompson, who was trained at the Banff Hospital, and subsequently engaged in nursing in San Francisco, U.S A., and elsewhere, has been appointed matron of the Turriff Hospital.

The City Hospital, Aberdeen.—Miss Margaret Fruter has been appointed Matron of the City Hospital, Aberdeen. She was trained at the Edinburgh Royal Infirmary, where she was nurse in the male medical wards, and night superintendent.

St. Olave's Union Infirmary.—Miss Mary Ann Sargeant has been appointed Matron of the above infirmary. She has held the posts of assistant nurse at the Woolwich Union Infirmary, nurse at the Bethnal Green Infirmary, head nurse at the Kington Union Infirmary, superintendent of nurses at the Hampstead Workhouse Infirmary, and superintendent nurse of the Camberwell Parish Workhouse Infirmary.

### Notes and Queries.

**Queries.**

(113) *New Quay.*—Will the Editor give the address of the boarding house at Newquay mentioned on July 4th?—*E. W.*

(114) *Unprofessional.*—Would it be unprofessional to send round a circular to inform my friends I am taking up private nursing?—*A. R.*

(115) *Infection.*—I want to go as probationer where no infectious cases are received.—*E. M. W.*

(116) *Spinal Carriage.*—Can you tell me where to apply for a spinal carriage for a poor child?—*E. G. H.*

(117) *Probationer.*—Would you put me in the way of obtaining information about children's hospitals?—*Rosemary.*

(118) *Sir Andrew Clark.*—Please inform me where I can learn particulars of the public and private life of Sir Andrew Clark.—*C. T. A.*

(119) *Cripple.*—Do you know of a home for incurables likely to receive a poor woman entirely crippled for from 2s. 6d. to 5s. weekly?—*Queen's Nurse.*

(120) *Training.*—Is the sixth standard training in a national school sufficient for a probationer to obtain a nursing certificate?—*M. L.*

(121) *Policy Holder.*—Where can I obtain three months' training without paying a fee?—*Midwifery.*

(122) *Roman Catholic.*—Where can a Roman Catholic receive training in a London hospital?—*A. M.*

(123) *Probationer.*—Where can a probationer be received without a fee?—*W. D*

(124) *Netley.*—Please advise me as to becoming an army sister.—*A. R.*

**Answers.**

(113) *New Quay (E. W. and others).*—So many inquiries have been received as to this that we cannot any longer send the written address, which is Mrs. North, Erncliffe, New Quay.

(114) *Unprofessional (A. R.).*—Certainly not. Nurses usually have a card which they enclose to friends—upon which her qualifications are briefly described, such as "Certificated Medical and Surgical Nurse."

(115) *Infection (E. M. W.).*—Very few hospitals now take infectious cases into the main buildings, but an epidemic may break out at any time, and it might be your duty to assist. We think you should hesitate to take up nursing if you cannot face infection.

(116) *Spinal Carriage (E. G. H.).*—By applying to the vicar of the parish, you might obtain a grant from the Hospital Sunday Fund. There is also the Surgical Aid Society. Possibly you might hire from one of the firms advertised in our columns. We have put your application in another column as well, hoping to assist you.

(117) *Probationer (Rosemary).*—You will find particulars of children's hospitals in "Burdett's Hospitals and Charities, 1896" (The Scientific Press).

(118) *Sir Andrew Clark (C. T. A.).*—No life of Sir Andrew Clark has been published. Good obituary notices appeared in the *British Medical Journal*, The Hospital, and many periodicals and newspapers at the date of his death. "Men of the Times" and the "National Dictionary of Biography" contain accounts of him.

(119) *Cripple (Queen's Nurse).*—The sum is too small, we fear, without subscriber's letter. We think advice and list of institutions given in "Burdett's Helps in Sickness and to Health" (Scientific Press) might help you.

(120) *Training (M. L.).*—Yes. Many wardmaids with this amount of education succeed as nurses.

(121) *Policy Holder (Midwifery).*—Write to the Secretary, Midwives' Institute, 10, Buckingham Street, Strand; she might kindly assist you.

(122) *Roman Catholic (A. M.).*—The London Hospitals receive nurses irrespective of creed, but as you desire to attend Mass on certain days you must apply stating this.

(123) *Probationer (W. D.).*—You will find a list of hospitals and particulars in "How to Become a Nurse" (Scientific Press). It is an advantage in such a case as you mention to act as wardmaid first. Watch our advertisement columns or advertise.

(124) *Netley (A. R.).*—Provincial training is quite sufficient provided it is in a large general hospital. Such training as you are receiving now will count for very little; you will need at least three years elsewhere to stand a good chance. Some fever training will be an advantage.

### Wants and Workers.

Wanted, a spinal carriage for a child whose parents are not in a position to purchase one. Reply to Nurse G. Heaton, 10, Kassala Road, Chelsea Reach, S.W.

## Figures and Tables

**Figure f1:**
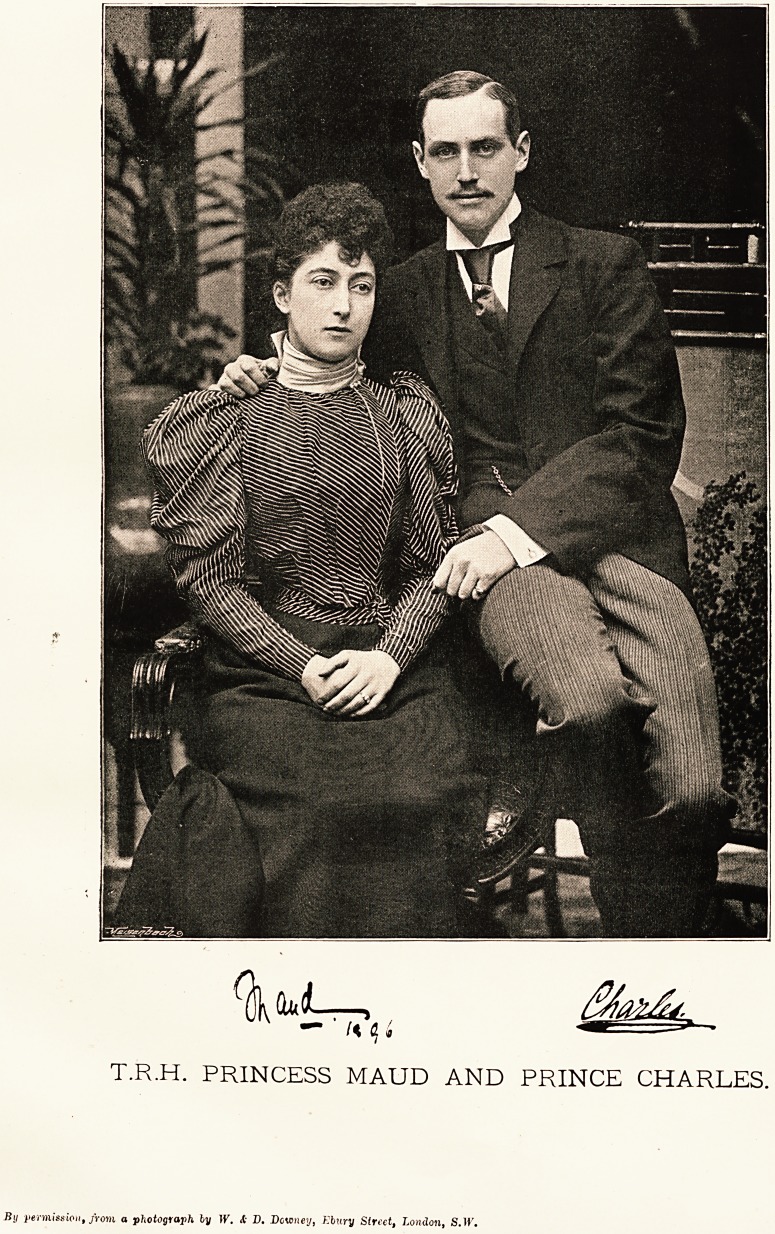


**Fig. 29. f2:**
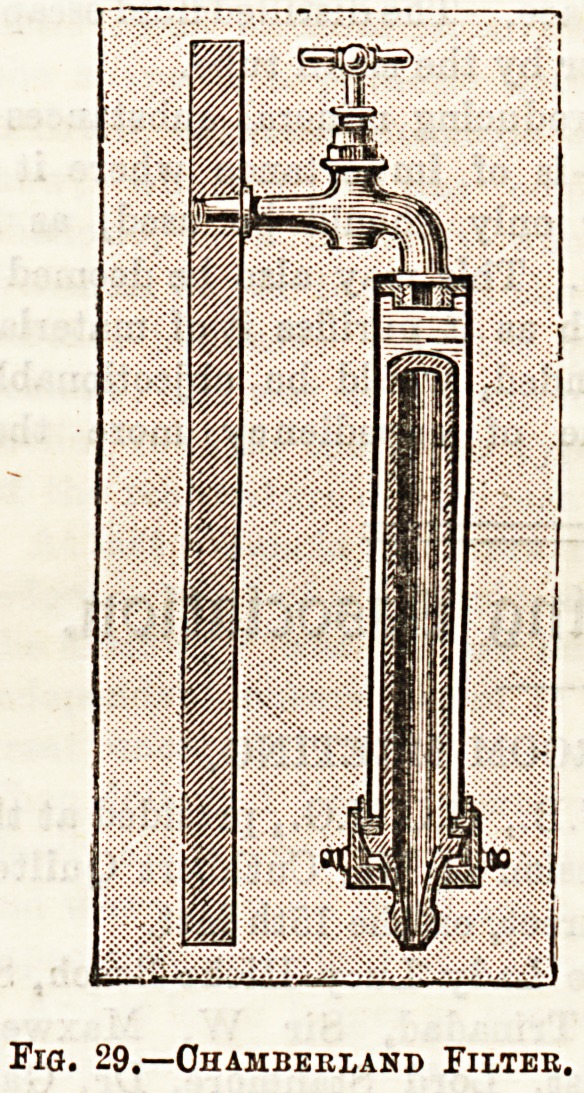


**Fig. 30. f3:**
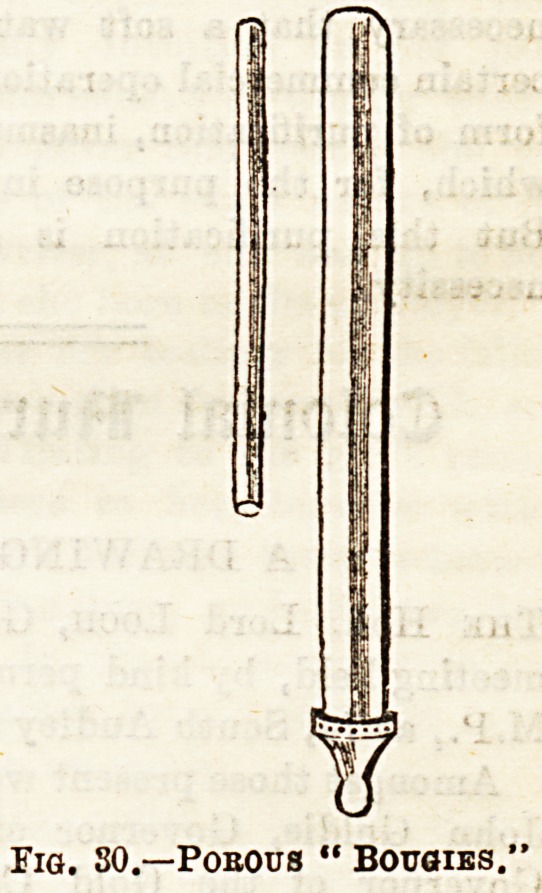


**Fig. 31. f4:**